# The prophylactic use of C1 inhibitor in hereditary angioedema patients undergoing invasive surgical procedures: a retrospective study

**DOI:** 10.1186/1710-1492-10-17

**Published:** 2014-04-23

**Authors:** Geneviève Gavigan, William H Yang, Stephanie Santucci, Rachel Harrison, Jacob Karsh

**Affiliations:** 1 Division of Dermatology, Department of Medicine, University of Ottawa, Ottawa, Canada; 2 Division of Dermatology, Department of Medicine, Ottawa Hospital Research Institute, Ottawa, Canada; 3 Allergy and Asthma Research Centre, Ottawa, Canada; 4 Division of General Internal Medicine, Department of Medicine, University of Ottawa, Ottawa, Canada

**Keywords:** Hereditary angioedema (HAE), C1 Inhibitor, Short-term prophylaxis (STP), Pre-procedural treatment

## Abstract

**Background:**

Hereditary Angioedema (HAE) is a rare autosomal dominant condition characterized by episodic angioedema, which may be triggered by invasive procedures and surgery. C1 inhibitor (C1 INH) was approved in the United States and Canada in 2009 and 2010, respectively, for the treatment of acute attacks. Most recently in April 2013, it was approved in Europe for short-term prophylaxis (STP), prior to medical, dental, or surgical procedures, to prevent HAE attacks in both children and adults. Currently, C1 INH is not approved in Canada or the United States for STP of HAE attacks. Our objective was to demonstrate the effectiveness of C1 INH as a short-term prophylactic treatment for patients with Type I HAE undergoing invasive surgical procedures.

**Methods:**

A retrospective chart review between 1997-2013 was performed at one Canadian Tertiary Care Allergy and Asthma Clinic affiliated with The Ottawa Hospital, in Ottawa, Canada. The standard dose of C1 INH for STP was 10 or 20 U/kg.

**Results:**

In all 24 procedures, there were no post-procedure HAE attacks after short-term prophylactic administration of C1 INH.

**Conclusions:**

In this retrospective chart review at one tertiary care Allergy and Clinical Immunology Clinic, short-term prophylactic use of C1 INH was found to be effective at preventing post-procedure HAE attacks, in patients diagnosed with Type I HAE.

## Background

Hereditary Angioedema (HAE) is a rare autosomal dominant disease with a prevalence of approximately 1 in 50,000 [1]. There are 3 variants of HAE, which present with episodic attacks of non-pitting and non-pruritic edema; however, they may be differentiated by CI INH level and function. Type I affects 85% of patients and is distinguished by low plasma levels of C1 INH; while in Type II, which affects 15% of patients, inactive C1 INH is produced, but plasma levels are normal. Type III is rare, primarily affects females, and is characterized by normal C1 INH level and function; it may be estrogen dependent [1].

Significant mortality is associated with HAE; undiagnosed HAE carries a 30-40% mortality rate, mainly from upper airway obstruction [2]. Furthermore, patients suffer from important morbidity and disabling symptoms, and may be debilitated for 20-100 days per year [3]. New medications have been developed for both treatment of acute attacks and for prophylaxis (short-term and long-term). Short-term prophylaxis (STP) is used to prevent attacks surrounding known triggers (surgeries, invasive dental procedures, stressful life events), while long-term prophylaxis is reserved for patients who suffer from frequent HAE attacks.

The World Allergy Organization (WAO) recognizes that physical and emotional stress associated with surgery may elicit HAE attacks. These attacks usually occur 4-30 hours post-surgery, and commonly near the site of physical trauma, suggesting that dental surgery may be associated with significant risk because of potential airway edema [4].

C1 INH has been marketed for the treatment of HAE attacks in Europe for over 30 years; it was officially approved in Europe for acute HAE attacks in 2008. It was approved in the United States (US) and Canada, in 2009 and 2010, respectively, for the treatment of moderate to severe acute abdominal and facial attacks. In April 2013, C1 INH was approved in Europe for STP in children and adults, prior to medical, dental, or surgical procedures. It is not currently approved for STP in Canada or the US. Our objective is to demonstrate the effectiveness of C1 INH as a short-term prophylactic treatment for patients with Type I HAE undergoing surgical procedures.

## Methods

A retrospective chart review was performed at one Canadian Tertiary Care Allergy and Clinical Immunology Clinic (Yang Medicine), affiliated with The Ottawa Hospital in Ottawa, Canada. The study included all patients with type I HAE who had received short-term prophylactic treatment with C1 INH (Berinert®) prior to an invasive procedure, surgery, dental work, or child birth, between 1997-2013. The diagnosis of Type I HAE was established in these patients based on clinical history, family history, and laboratory values (C4 level, C1 INH level). The primary outcome was the number of post-procedural HAE attacks after short-term prophylactic administration of C1 INH.

The standard dose for C1 INH (Berinert®) was either 10 U/kg or 20 U/kg. This range in dosing can be explained by to the long duration of the study (1997-2013). Prior to the IMPACT-1 trial in 2009, the dose of 10 U/kg was used as standard; however after the IMPACT-1 trial found that 20 U/kg was superior to 10 U/kg [5], this dose was utilized instead.

Prior to 2010 (when Berinert® was approved in Canada for acute attacks), the medication was only accessible through Health Canada’s Special Access Programme on a case-by-case basis. It was requested for each HAE patient on the basis of the possible serious and potential life-threatening nature of an HAE episode caused by an invasive procedure or surgery.

## Results

At total of 24 surgical procedures (Table 1) were performed on 12 patients (4 male, 8 female) with Type I HAE. The average number of procedures per patient was 2 (range 1-6), and the average age at the time of the procedure was 42 (range 19-62).

**Table 1:** Procedure breakdown

**Procedure characteristics**					**Patient characteristics**		
**#**	**Year**	**Description**	**C1 INH dose Received (U)**	**Timing of C1 INH administration**	**Gender**	**Age**	**Concomitant long–term treatment for HAE**
1	2013	Cesarian Section	2000 U	Just prior to procedure	Female	28	-
2	2013	Dental extraction	2000 U	Just prior to procedure	Male	49	-
3	2013	Vaginal child birth	1500 U	Prior to delivery, while in active labour	Female	26	-
4	2012	Inguinal hernia repair	1000 U/h × 3h	For the length of the surgery (3h)	Male	49	-
5	2012	Dental surgery	1500 U	Just prior to procedure	Female	62	-
6	2012	Port-o-cath placement	1500 U	Just prior to procedure	Female	27	-
7	2011	Port-o-cath placement	1500 U	Just prior to procedure	Female	26	-
8	2011	Hickman catheter placement	1500 U	Just prior to procedure	Female	25	-
9	2011	Vaginal child birth	1500 U	Prior to delivery, while in active labour	Female	33	-
10	2010	Dental extraction	1000 U	Just prior to procedure	Male	57	Danazol
11	2010	Dental extraction	1000 U	Just prior to procedure	Male	47	Danazol
12	2007	Dental surgery	1000 U	Just prior to procedure	Female	58	-
13	2006	Hernia repair	1000 U	Just prior to procedure	Female	46	-
14	2006	Dental extraction	1000 U	Just prior to procedure	Male	53	Danazol
15	2005	Dental extraction	1000 U	Just prior to procedure	Female	19	-
16	2004	Abdominal aneurysm repair	1500 U	Just prior to procedure	Male	51	Danazol
17	2003	Excision of melanoma-in-situ	1000 U	Just prior to procedure	Female	53	-
18	2003	Angiogram	1500 U	Just prior to procedure	Male	49	Danazol
19	2001	Liver biopsy	1500 U	Just prior to procedure	Male	38	Danazol
20	2000	Ascending and descending aortic aneurysm repair	3000 U	Just prior to procedure	Male	47	Danazol
21	1998	Angiogram	1000 U	Just prior to procedure	Female	55	-
22	1998	Hysterectomy	1500 U	Just prior to procedure	Female	38	-
23	1997	Dental surgery	1000 U	Just prior to procedure	Female	39	-
24	1997	Aortic valve replacement	1500 U	Just prior to procedure	Male	44	Danazol

The majority of surgical procedures (22/24) were treated with a short-term prophylactic C1 INH dose of 1000 U, 1500 U, or 2000 U, which corresponded to a dose of 10 or 20 U/kg, administered IV over a 20-30 minute period just before the procedure. Procedure 4 was treated with an infusion of C1 INH, resulting in a higher cumulative dose. This decision was based on mutiple factors, including the invasiveness of the surgery, the need for intubation, the patient’s history of a possible previous laryngeal attack, and the patient being off his Danazol at the time of the surgery. Procedure 20 also received a higher prophylactic dose of C1 INH (3000 U), because of the seriousness and length of the surgery.

In eight of the procedures, the patient received STP with C1 INH in addition to long-term treatment with Danazol. STP with C1 INH was provided to these patients as they continued to experience HAE attacks while taking Danazol.

In all 24 surgical procedures, there were no post-procedure HAE attacks after short-term prophylactic administration of C1 INH. Furthermore, no patient required additional dosing because of concern regarding prodromal symptoms of an impending HAE attack.

## Discussion

In our experience, C1 INH was an effective short-term prophylactic treatment for Type I HAE patients undergoing invasive procedures. There were no post-procedural HAE attacks following any of the 24 procedures. Our results are consistent with a previous report by Grant et al, where pre-procedural nanofiltered C1 INH (Cinryze®) was used to prevent HAE attacks. In that study, 41 patients (33 adults, 8 children) received C1 INH prior to 91 procedures (87/91 procedures were treated with a dose of 1000 U for STP). No HAE attack followed 89 of 91 procedures; the 2 attacks were successfully treated with 1000 U of C1 INH [6]. It is noteworthy, however, that the majority of the procedures in the study by Grant et al did not require pretreatment.

Surgery is a recognized precipitant of HAE attacks [4]. Bork et al. observed 124 attacks (88 facial, 8 laryngeal, 28 combined) following 577 dental extractions without prophylaxis [7]. Even with STP, HAE attacks are not completely eliminated. Farkas et al. reported 13 HAE attacks following 134 surgical procedures despite prophylaxis: 5/38 with Danazol, 3/9 with Tranexamic acid, and 5/87 with 500 U of C1 INH (Berinert®) [8]. Different short-term prophylactic doses of C1 INH (Berinert®) were assessed for dental extraction; 12 attacks followed 75 extractions after 500 U, and 4 attacks followed 53 extractions despite 1000 U [7].

While the studies by Grant et al, Bork et al, and Farkas et al each observed post-procedural HAE attacks despite STP with C1 INH, our study found no attacks. Several factors may contribute to this, including the sample size in our study being smaller than in the others. In addition, the doses of C1 INH used in our study (standard dose of 10 U/kg, which was increased to 20 U/kg after the IMPACT-1 trial in 2009) corresponded to a dose of 1000 U, 1500 U, or 2000 U for most patients. Thus, for 15/24 procedures, patients received doses greater than 1000 U, which was greater than doses used by Grant et al (in 87/91 patients) and Bork et al. [6,7] and for all 24/24 procedures, patients in our study received doses greater than the dose of 500 U employed by Farkas et al. [8].

Historically, treatment options for HAE have included stimulating endogenous C1 INH synthesis using attenuated androgens, reducing C1 INH consumption with protease inhibitors (e.g. transexamic acid), and replacing C1 INH with fresh frozen plasma or C1 INH (plasma derived or recombinant); plasma derived C1 INH being the prefered option for acute attacks for the last 30 years [9]. Recombinant C1 INH has also been investigated for its use as a prophylactic treatment and prelimary results may be promising; [10] however, the half-life for recombinant C1 INH is shorter than that of plasma derived, which may limit its effectiveness for prophylaxis and more data are needed before commencing this therapy. Newer medications are approved for the treatment of acute attacks, STP, or long-term prophylaxis. Tables 2, 3, and 4, present the approved treatments in Canada, the United States, and Europe for the treatement of acute HAE attacks, short-term prophylaxis of HAE attacks, and long-term prophylaxis of HAE attacks, respectively.

**Table 2:** Medications approved for the treatment of acute HAE attacks

**Treatment of acute attacks**					
**Medication class**	**Example**	**Dose**	**Approval**		
			**Canada**	**US**	**Europe**
**C1 INH Replacement (plasma derived)**	Berinert	20 U/kg IV	✓	✓	✓
	Cetor	1000 U IV			✓
	Cinryze	1000 U IV			✓
**Recombinant C1 INH**	Ruconest	50 U/kg IV			✓
	Rhucin	50 U/kg IV		Pending	
**Kallikrein Inhibitor**	Ecallantide	30 mg SC		✓	
**Bradykinin antagonist**	Icatibant	30 mg SC	Pending	✓	✓

**Table 3:** Medications approved for short-term prophylaxis of HAE attacks

**Short-term prophylaxis**					
**Medication class**	**Example**	**Dose**	**Approval**		
			**Canada**	**US**	**Europe**
**C1 INH replacement (plasma derived)**	Berinert	10 to 20 U/kg body weight or 1000 U IV*			✓
	Cinryze	500-1500 U IV 1 hour prior to event		✓	✓
**Attenuated androgens**	Danazol	200 mg PO TID × 5-7 d before procedure, and 4 d post-procedure		✓	

Of note: androgens are contraindicated in: pregnancy, lactation, and growing children. They are also associated with severe side-effects.

*Recommendation varies as dose yet to be fully investigated.

**Table 4:** Medications approved for long-term prophylaxis of HAE attacks

**Long-term prophylaxis**					
**Medication class**	**Example**	**Dose**	**Approval**		
			**Canada**	**US**	**Europe**
**C1 INH replacement (plasma derived)**	Cinryze	1000 U IV every 3-4 days	✓	✓	✓
**Attenuated androgens**	Danazol	≦ 200 mg PO TID		✓	

Of note: androgens are contraindicated in: pregnancy, lactation, and growing children. They are also associated with severe side-effects.

The 2010 International consensus algorithm for the diagnosis, therapy and management of HAE provided guidelines for STP, suggesting that it is not routinely required before minor manipulations (e.g. minor dental work), so long as C1 INH is immediately available and the manipulation has not previously triggered an attack. For major procedures or intubation, they recommended short-term prophylactic C1 INH 1-6 hours before the procedure [11]. Similarly, the WAO urges considering prophylactic C1 INH 1-6 hours before surgeries, especially dental and intraoral, those requiring endotracheal intubation, those manipulating the upper airway or pharynx, and for endoscopy and bronchoscopy procedures [4]. Both groups acknowledged that the optimal dose of C1 INH is unknown, but suggest a dose of 10-20 U/kg [4,11], or 1000 U [4]. Finally, the US Hereditary Angioedema Association Medical Advisory Board 2013 recommended that C1 INH for STP be administered 1-12 hours before the stressor; alternatively, anabolic steroids given 7-10 days before the stressor may be provided for STP. The importance of having on-demand treatment available, regardless of whether the patient was given STP for the stressor or not, was strongly emphasized [12].

## Conclusions

We have demonstrated successful use of C1 INH as STP for Type I HAE patients undergoing invasive procedures. Our study was limited by our small sample size, the retrospective design, and only assessing a single agent, C1 INH. Other agents, such as attenuated androgens, fresh frozen plasma, and transexamic acid were not specifically included in our study. Although future prospective trials are needed, data suggest that pre-procedural treatment with C1 INH, Berinert®, may be an effective to substantially decrease post-procedural HAE attacks.

## Abbreviations

C1 INH: C1 inhibitor; HAE: Hereditary Angioedema; STP: Short-term prophylaxis; US: United States; WAO: World Allergy Organization.

## Competing interests

GG: No competing interests to declare. WHY: CSL Behring National Advisory Board member, received honorarium as speaker, received research grant for HAE studies for acute attacks, not for prophylaxis. SS: No competing interests to declare. RH: No competing interests to declare. JK: No competing interests to declare.

## Authors’ contributions

WHY created the study, and participated in its design and coordination and helped to draft the manuscript. All the patients in this study are patients of WHY. SS is the head nurse and coordinator in for HAE patients in WHY’s allergy clinic. SS communicated with all the patients, collected data, and helped draft the manuscript. GG organized, analyzed, and interpreted all of the data, and was the principal author in drafting the manuscript. RH assisted in drafting the manuscript, and collected data for the manuscript. JK assisted in the manuscript overall design and journal selection. All authors read and approved the final manuscript.
